# Identification of IspD as a novel target for tuberculosis treatment using compound M6

**DOI:** 10.3389/fmicb.2024.1461227

**Published:** 2024-11-13

**Authors:** Lijun Dong, Hui Qi, Yue Zhu, Yuma Yang, Yue Zhao, Sihan Zhang, Yongqiang Su, Taiyun Yue, Xiancai Du, Hetian Lei, Yanhui Yang

**Affiliations:** ^1^Ningxia Key Laboratory of Clinical and Pathogenic Microbiology, The School of Basic Medical Sciences, Ningxia Medical University, Yinchuan, China; ^2^Shenzhen Eye Hospital, Jinan University, Shenzhen Eye Institute, Shenzhen, China; ^3^School of Medicine, Southern University of Science and Technology, Shenzhen, China; ^4^Department of Ophthalmology, The First People’s Hospital of Chenzhou, Chenzhou, China

**Keywords:** *Mycobacterium tuberculosis*, molecular docking, N-(1, 3, 4-oxazole-2-)-benzolamide, IspD, CRISPR/Cas9

## Abstract

**Introduction:**

Tuberculosis (TB) is a serious infectious disease that endangers human health, and TB becomes more difficult in eradiation due to its multidrug resistance (MDR). The objective of this research was to identify novel targets for treating TB.

**Methods:**

A 2-fold serial dilution method was used to determine minimal inhibitory concentrations (MIC) of compound M6 against *Mycobacterium smegmatis* (*M. smegmatis*). Compound M6 was subjected to reverse molecular docking with seven *Mycobacterium tuberculosis* proteins, and the best binding protein with the highest LibDock score was evaluated. The target protein with the highest score was purified through prokaryotic expression. Isolated target proteins were investigated for the enzyme activities and for the kinetic effect of compound M6 by absorbance detection. Subsequently, the CRISPR/Cas9 technology was employed to inhibit target gene expression for detecting MIC changes. Finally, potential targets were evaluated for the effect of the compound M6 in bacteria.

**Results:**

The MIC values of compound M6 against *M. smegmatis* were 32 μg/mL. The results from reverse molecular docking show that IspD has the highest LibDock score of 142.50, followed by Rv0674, IspF, and Dxr, with docking scores of 110.762, 71.6955, and 57.7446, respectively. IspD is a key enzyme in the 2-C-methyl-D-erythritol 4-phosphate pathway of MTB. The aKi and Ki values of M6 for the substrate MEP are 609.58 μM and 81.33 μM. For CTP, the aKi and Ki values are 657.89 μM and 40.07 μM. With tetracycline inducing CRISPR/Cas9 to suppress the expression of IspD, the MIC value of M6 against IspD went down significantly from 32 μg/mL to 4 μg/mL.

**Conclusion:**

IspD is a novel target of the compound M6 for treating TB.

## 1 Introduction

Tuberculosis (TB) is a noteworthy infectious disease caused by *Mycobacterium tuberculosis* (*MTB*) that threatens human health worldwide (Howard and Khader, 2020). In 2022 alone, TB caused 1.30 million deaths and approximately 10.60 million new cases of TB. The number of new patients with rifampicin-resistant tuberculosis (RR-TB) was 45 (95% CI: 39.9–50.1) million (WHO, 2021). In recent years, multidrug resistant tuberculosis (MDR-TB) and the co-infection of TB with HIV have increased the difficulty of treating TB, with a serious decline in cure rates (Shapiro and Ross, 2021; Kohli and Schiller, 2021). MDR-TB is the most common form of drug-resistant tuberculosis, a disease caused by strains of *Mycobacterium tuberculosis* that are resistant to at least isoniazid and rifampin, the two most effective anti-tuberculosis drugs. The probability of emergence of new drug-resistant TB increases with prolonged treatment duration (Gatadi and Nanduri, 2020; Miotto and Zhang, 2018). Thereby, it now is urgent to discover novel drugs for the treatment of *MTB*.

We have found multiple compounds with an anti-*MTB* activity *in vitro* through phenotypic screening of *MTB H37Rv* strain, in which the MIC of N-(1, 3, 4-oxazole-2-)-benzolamide compound (M6) against *MTB H37Rv* was 0.5 μg/mL. The MIC of clinically isolated and drug-resistant *MTB* was 1.0 μg/mL, suggesting that there is possibly no cross-resistance between M6 and existing anti-tuberculosis drugs, and the molecular mechanism of M6 is probable to be original. M6 has a weak cytotoxicity, and the semi-inhibitory concentration of IC_50_ values for Hela cells was about 20 μg/mL, which was 20–40 times higher than the MIC values of M6 against *MTB H37Rv*. The structure of M6 is different from that of anti-tuberculosis drugs in clinical applications and it is in an under-development stage. Therefore, M6 is a potential new TB drug. In addition, it is useful for discovering potential novel drug targets (Gao and Yang, 2012).


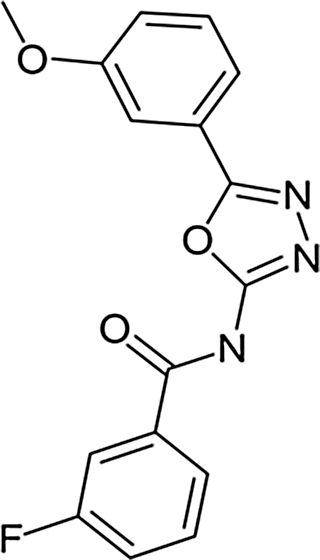


Molecular docking is a highly effective computational method for exploring the interactions between molecules, specifically studying how ligands bind with proteins through computer simulation techniques (Ferreira et al., 2015). In the past, popular molecular docking tools like AutoDock (Eberhardt et al., 2021), FlexX (Schellhammer and Rarey, 2004), and GOLD (Jones et al., 1997) have played a crucial role in the domain of drug screening. As bacterial resistance continues to evolve, designing new drugs that specifically bind to relevant biological targets remains a common task for medicinal chemists. The reverse docking protocol can be utilized to identify novel protein targets for drugs, propose new mechanisms of action, and also to discover new therapeutic uses for existing drugs (Caballero, 2021). Several studies have successfully employed molecular docking to investigate the interactions between natural molecular compounds and specific protein targets (Meng et al., 2011; Seyedi et al., 2016), such as the study by Ali MT et al. on the anti-tuberculosis potential of propolis constituents (Ali et al., 2018).

In the present study, the optimal binding molecule was found to be IspD using reverse docking of the known active compound M6 with the receptor biomolecule. IspD, also named as 2-C-methyl-ᴅ-erythritol 4-phosphate cytidylyltransferase, is a key enzyme of the 2-C-methyl-ᴅ-erythritol 4-phosphate (MEP) pathway (Testa and Brown, 2003; Hsieh and Chang, 2008; Bartee and Wheadon, 2018). In *MTB*, isoprenoids and their derivatives, which are the end products of the MEP pathway (Gao and Yang, 2012), are essential precursors for bacterial metabolism and cell wall synthesis. In addition, the MEP pathway is deficient in human and animal, so IspD has the potential to be an anti-TB drug target with advantages of excellent specificity (Eoh and Brown, 2007).

## 2 Materials and methods

### 2.1 Major reagents and materials

The *Escherichia coli* receptor bacteria were purchased from Quan-Shi-Jin Biological Co., Ltd. (Beijing, China). *Mycobacterium smegmatis* MC^2^155 were a generous gift from Professor Xiao Chunling from the Institute of Medical Biotechnology of the Chinese Academy of Medical Sciences. Plasmid pET28a (+) was stored in this experiment, and plasmids pRH2521 (Cat. 84380) and pRH2502 (Cat. 84379) were purchased from Addgene (Cambridge, MA). Various media: 7H9, 7H10, Middlebrook OADC, BHI, and BHIA were purchased from BD Biosciences (Woburn, MA). Endonuclease MluI, BbsI, and EcoRI were purchased from New England Biolabs (Boston, MA). Takara TB Green Premix Ex Taq II, RNaseH Plus, and Prime Script RT Master Mix were ordered from Takara (San Jose, CA). Other reagents were purchased from Yinchuan Weiboxin Biotechnology Co., Ltd. (Yinchuan, China). An antibody against the Isocitrate dehydrogenase (ICD) were a generous gift from Professor Zhang Xuelian from the Institute of State Key Laboratory of Genetic Engineering, School of Life Sciences, Fudan University, and another antibody against IspD protein is the polyclonal antibody prepared by our research group themselves.

### 2.2 The MIC values of M6 against bacteria

To study the minimum inhibitory concentration (MIC) values of M6 against various bacteria using gradient dilution, we tested the following bacterial strains: *Escherichia coli, Pseudomonas aeruginosa, Klebsiella pneumoniae, Mycobacterium smegmatis, Enterococcus faecalis, Proteus* spp.*, Staphylococcus aureus, Staphylococcus epidermidis, Bacillus subtilis, Corynebacterium glutamicum, and Mycobacterium tuberculosis. Mycobacterium smegmatis* and other bacteria were cultured to a log phase and then spread onto 96-well plate. The highest concentration of M6 within the medium was 256 μg/mL and then diluted to 0.5 ng/mL by the double dilution method. The bacteria were cultured at 37°C to the stationary stage, and 100 μg/mL of the resazurin sodium salt solution was added. The OD_600_ values for each well were measured on a microplate reader (Patel and Lubanski, 2009; Lepe and Domínguez-Herrera, 2014).

### 2.3 Molecular docking

Discovery Studio is a visualization tool that integrates the storage and management of experimental data through the use of modeling and simulation toolsets, facilitating the viewing and analysis of proteins and modeling data (Dong et al., 2021). We employed Discovery Studio 2.5 LibDock to carry out molecular docking of proteins Rv0674, CoaA, IspD, Dxr, IspC, IspE, and IspF with compound M6. The docking outcomes and the conformational integration of the small molecule with the proteins were analyzed to select the protein with the best binding affinity for expression and inhibition characteristic studies (Karakkadparambil Sankaran and Nair, 2023).

### 2.4 Expression of IspD proteins in bacteria

Based on the *M. smegmatis* IspD gene sequence (*M. smegmatis* MEI_5916) in NCBI, a plasmid pET28a inserted with ispD was synthesized by Tsingke Biotechnology Co., Ltd. This recombinant plasmid was transformed into *E. coli* BL21 (DE3) receptor bacteria, which were cultured and then induced with 1 mM IPTG at 16°C with 180 rpm for 14 h. The bacterial lysates were clarified by centrifugation at 4°C, 8,000 *g* for 30 min, and the supernatant was subjected to His trap TM HP affinity chromatography column by gravity. This column was then washed by imidazole eluents (the concentrations of imidazole: 5 mM, 10 mM, 20 mM, 50 mM, 100 mM, 200 mM, and 500 mM), and 10 μL of each solution was collected for 10% SDS-PAGE and Coomassie bright blue staining (Li and Gao, 2011).

### 2.5 Mechanical analysis of IspD enzyme activity

The IspD of *MTB H37Rv* specifically catalyzes transformation of 2-C-methyl-D-erythritol 4-phosphate (MEP) by the energy of Cytidine-5′-triphosphate (CTP) to form Cytidine-5′-diphosphate-2-C-methyl-D-erythritol (CDP-ME). The formation of one mole of CDP-ME concurrently results in the release of one mole of pyrophosphate (PPi) (Allamand and Iechowiak, 2023). PPi can react with ammonium molybdate solution to form a blue complex with a maximum absorbance observed at 590 nm. Therefore, the rate of formation of the blue complex can be used to determine enzyme activity and the extent of the reaction. A standard enzyme reaction system contains Tris-HCl (50 mM, pH 7.8), MEP (250 μM, 500 μM), CTP (250 μM, 500 μM), NaF (10 mM), MgCl (10 mM), DTT (10 mM), M6 (62.5 μM, 125 μM, 250 μM, 500 μM), with a total volume of 100 μL. The mixed reactants were incubated at 37°C for 40 min. Then, 10 μL of *β*-mercaptoethanol and 40 μL of ammonium molybdate were added, and the reaction was terminated after incubating at 37°C for an additional 20 min. Finally, the light absorption value of the reaction system was detected at 590 nm using an enzyme label. The inhibitory type of M6 as an inhibitor was determined by the double reciprocal method, and the inhibitory constant *K_i_* and α*K_i_* were calculated.


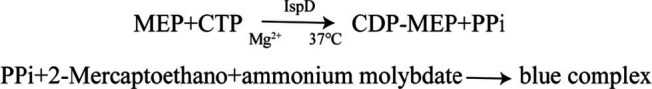


### 2.6 Depletion of IspD in *Mycobacterium smegmatis* using CRISPRi

Oligos for sgRNA-ispD (5′GCCGAAAGCATTCGTGACACT3′) and sgRNA–hif1A (5′GAAGTGTACCCTAACTAGCC3′) were cloned into the plasmid pRH2521 by BbsI. Human hif-1a was used as a negative control. The plasmid pRH2521-ispD, pRH2521-hif-1A, and a plasmid pRH2502 were transformed into *M. smegmatis* competent cells by electro transformation using a Bio-Rad electro converter (2.5 kv, 25 μF, 1,000 *Ω*). The resulting colonies were screened by double resistant plates with hygomycin and kanamycin (Singh and Carette, 2016).


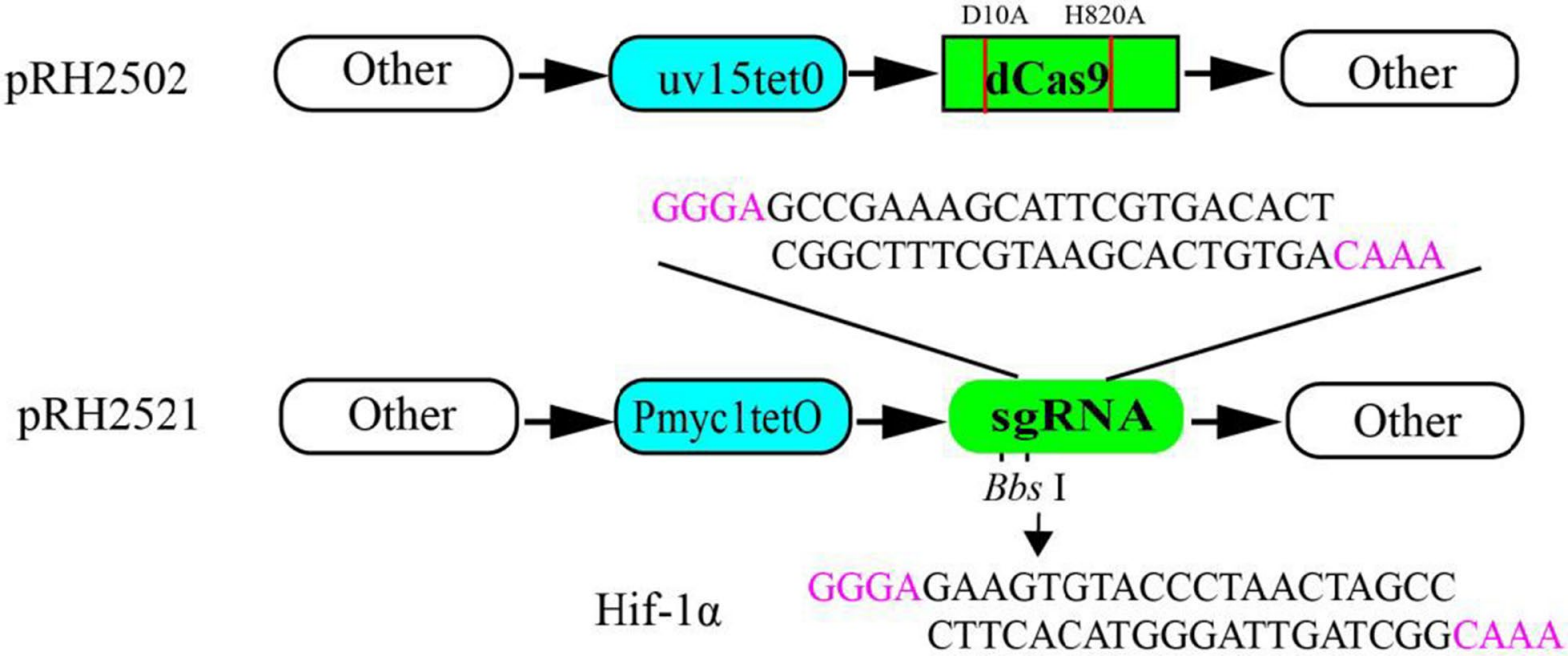


### 2.7 Treatment of the *Mycobacterium smegmatis* strain expressing IspD with M6

The *M. smegmatis* strain with IspD depletion and control colonies were cultured in at 37°C, 200 r.p.m. for 2 days, respectively. The bacterial solution was diluted at a ratio of 1: 1,000 supplemented with 100 ng/mL of anhydrous tetracycline (ATC). The MIC of M6 against different strains was measured in *M. smegmatis* with IspD depletion and control as described above. Single colony was selected in the 7H9 medium at 37°C, 200 rpm to logarithmic growth stage (OD_600_ = 0.6–0.8). A 1 mL bacterial solution was centrifuged to collect bacteria for phenotypic determination.

### 2.8 Determination of bacterial growth phenotype

*Mycobacterium smegmatis* strain with IspD inhibition was cultured until the OD_600_ reaches 0.6, and 1 mL culture was taken for centrifugation to collect the bacteria. The resultant pellets were re-suspended in 100 μL of 7H9-OADC media, which were diluted gradually to 10^−12^. In the control and pRH2521-ispD groups, 3 μL of each gradient were, respectively, placed on the double resistant 7H10-OADC solid plate and the double resistant 7H10-OADC solid plate containing 300 ng/mL ATC. 1% of these re-suspended bacteria was added into 10 mL 7H9-OADC medium, and this solution was cultured at 37°C, 180 r.p.m. for 6 h, and then 100 ng/mL, 200 ng/mL, and 300 ng/mL ATC was added for a shock culture. After inoculation for 6 h, 12 h, 18 h, 24 h, 30 h, 36 h, 42 h, 48 h, 54 h, and 70 h, samples were taken for measuring enzyme markers at 450 nm. Three parallel controls were set for each trial and the growth curves were plotted.

### 2.9 Quantitative PCR and Western blot

The genome of *M. smegmatis* was used for fluorescence qPCR with a Takara TB Green PremixEx Taq II (Tli RNaseH Plus) kit. When positive colonies of *M. smegmatis* were cultured to 0.2–0.3 (OD_600_), 100 ng/mL anhydrous tetracycline was added, and the culture was continued until the logarithmic growth stage. Then their RNA was extracted using the OMEGA Bacterial RNA Kit (R6950) kit for the reverse transcription reaction using the Prime Script RT Master Mix kit (Takara). The cDNA obtained was subjected to fluorescence qPCR using the Takara TB Green Premix Ex Taq II (Tli RNaseH Plus) kit (Qi and Dong, 2022). The qPCR data were combined with the standard curve.

The positive colonies were cultured to 0.6 (OD_600_), the bacteria were collected by centrifugation with 1 mL cultured medium, and the bacteria were re-suspended in 100 μL 7H9-OADC medium, then added to the original 7H9 medium by one in a thousand, cultured at 37°C, 180 r.p.m. for 3–4 h, then 100 μg/mL anhydrous tetracyclines were added respectively, and cultured under the same conditions for 48 h. Centrifuge was at room temperature 8,000 r.p.m., ultrasonic crushing bacteria in ice water bath, centrifuge at 4°C, 1,2000 r.p.m. for 30 min, and take supernatant. All the supernatant was added into a 3 kDa ultrafiltration tube at 4°C, 5,000 r.p.m., and centrifuged until 1 mL of liquid remained. The concentration of total protein was determined using the BCA method. 30 μg whole bacterial protein was taken for Western blot assay. The first antibody was a mouse polyclonal antibody to *M. smegmatis* IspD, the internal reference, and the second antibody was a mouse anti-IGI labeled with horseradish peroxidase. The expression for the IspD was calculated by Image J (Qi and Dong, 2022).

### 2.10 Quantitative PCR and Western blot

Samples under each treatment condition were subjected to three independent experiments. All data are presented as the means ± standard deviation (SD). The differences between groups were estimated by one-way analysis and a value of *p* < 0.05 was considered to indicate astatistically significant difference.

## 3 Results

### 3.1 M6 functions against various bacteria

**Figure 1:**
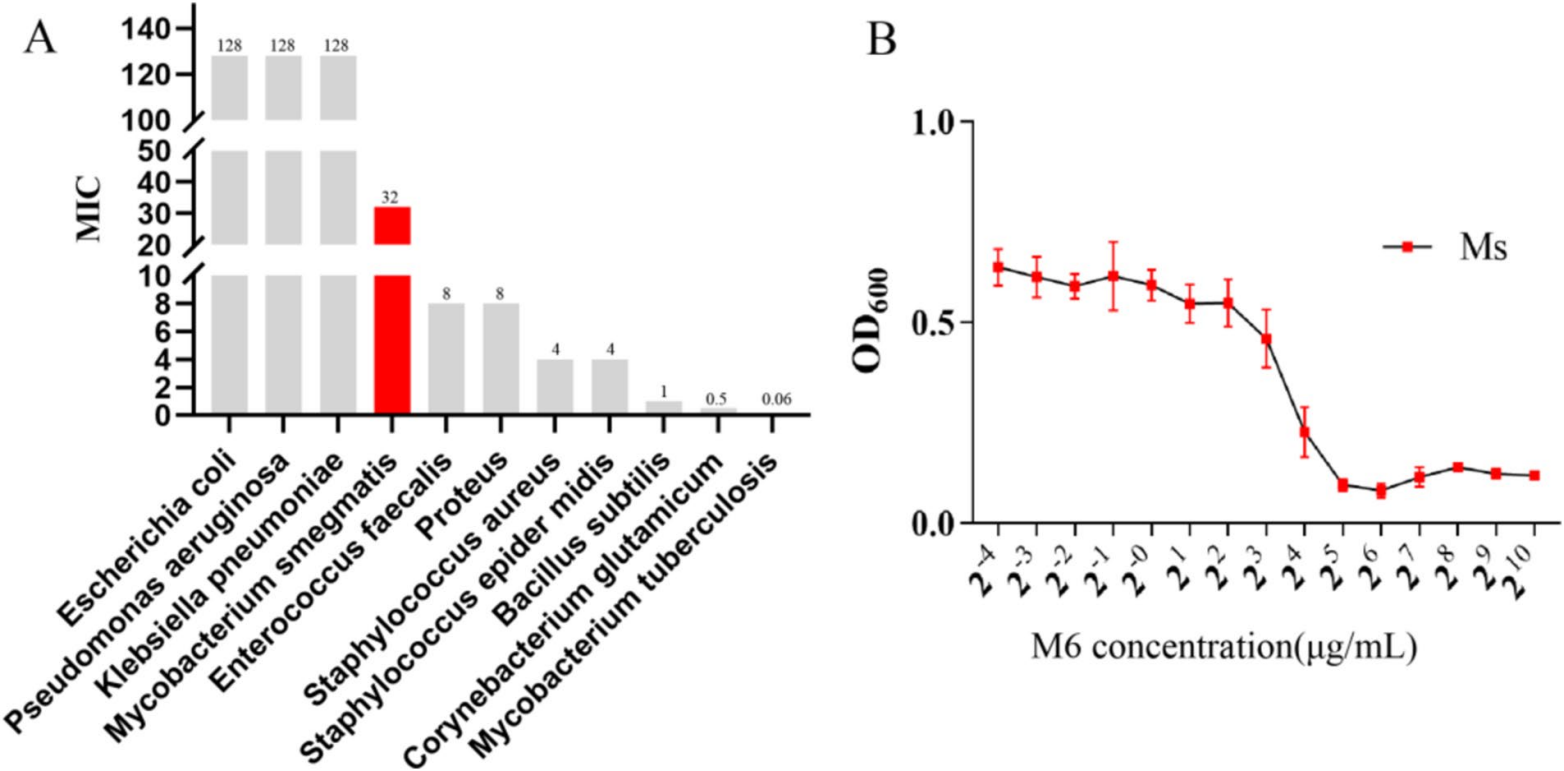
M6 functions against bacteria: **(A)** M6 MIC values for each bacterium. **(B)** Growth curves for *Mycobacterium smegmatis* at different M6 concentrations.

To determine if M6 could function against bacteria, M6 was added to bacterial culture in 96-well plates and their harvests were used to analyze the absorbance of OD_600_ with a microplate reader. The vertical coordinate in Figure 1 represented the absorbance of OD_600_, while the horizontal coordinate indicated the concentration of M6. The survival curve was used to calculate the minimum inhibitory concentration (MIC) value of M6 against each bacterium (Figure 1A). The results (Figure 1B) showed that MIC of compound M6 on *M. smegmatis* were 32 μg/mL.

### 3.2 Screening novel drug targets: reverse docking of M6 with proteins

In our study, we conducted reverse molecular docking of M6 compound with seven *Mycobacterium tuberculosis* proteins and evaluated the optimal binding protein with the highest LibDock score to identify and confirm candidate targets for drug-resistant TB. The protein IspD achieved the highest LibDock score of 142.50, followed by Rv0674, IspF, and Dxr with dock score values of 110.762, 71.6955, and 57.7446, respectively. However, IspC, IspE, and CoaA did not have any associated ligands (Table 1).

**Table 1:** Molecular docking Libdock scoring table.

Protein molecules	Libdock	poses	Protein name
2xwn	122.854	10 poses	IspD
3p2m	110.762	10 poses	Rv0674
3Q8H	71.6955	One pose	IspF
3zhy	57.7446	One pose	Dxr
1onp	No ligands docked		IspC
2v34	No ligands docked		IspE
2get	No ligands docked		CoaA

### 3.3 IspD of *Mycobacterium smegmatis* can be purified *in vitro*

**Figure 2:**
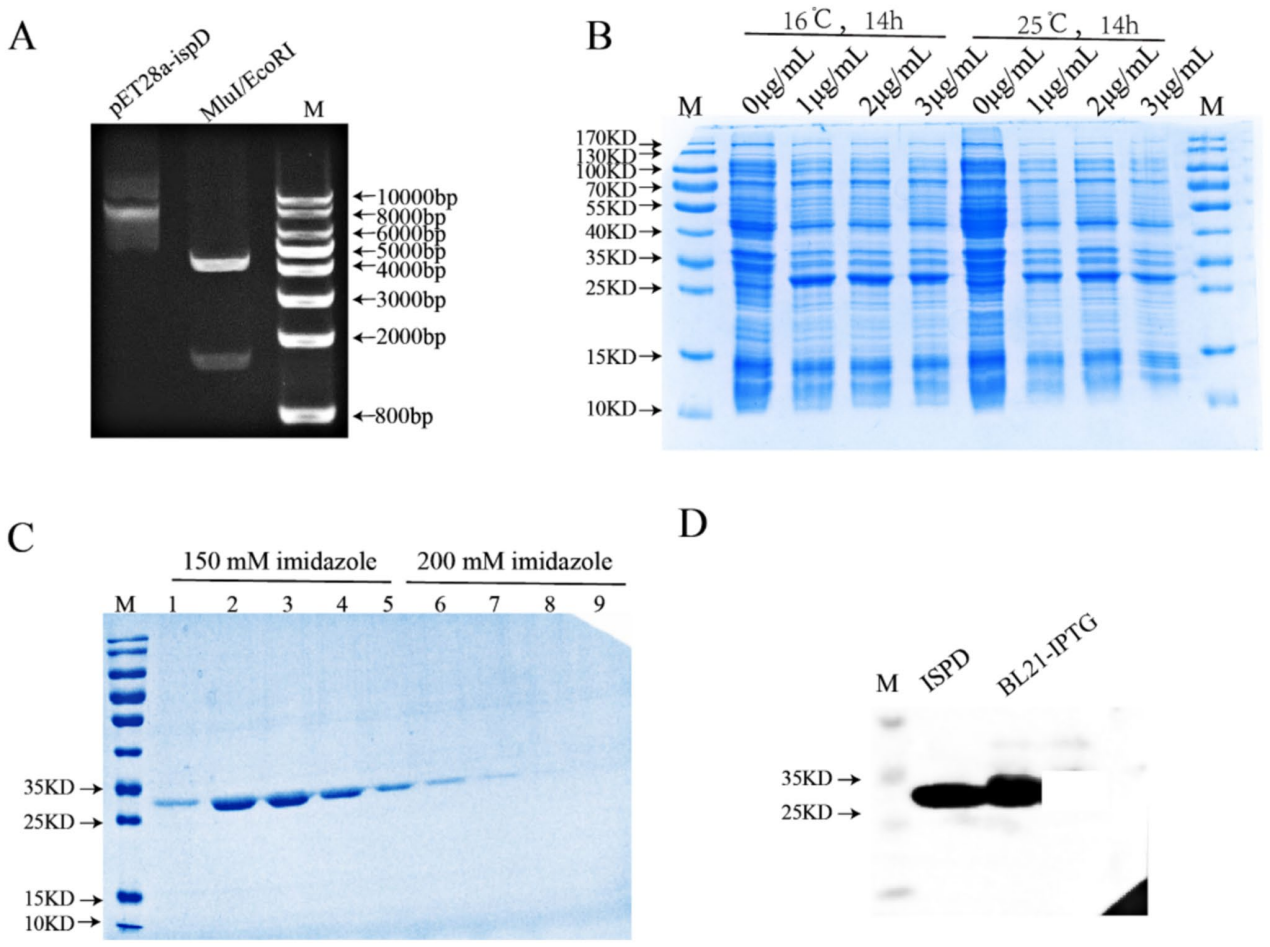
*In vitro* expression and purification of IspD protein. **(A)** pET28a-ispD was verified by double enzyme digestion of *Mlu*I and *EcoR*I. **(B)** Exploring induction conditions *in vitro* for expression of recombinant IspD proteins. **(C)** Coomassie blue staining of purified recombinant IspD protein. **(D)** Western blot analysis of recombinant IspD protein.

We next would explore the function of IspD, thereby the first thing we attempted to do was to purify IspD. Thus, the IspD gene from *M. smegmatis* was cloned into pET28a-ispD using MluI and EcoRI restriction enzymes (Figure 2A). The resulting construct was transformed into BL21 (DE3) bacteria and induced with IPTG at 16°C for 14 h (Figure 2B). As depicted in Figure 2C, a protein with a molecular weight of approximately 26 kDa was isolated using His trapTM HP affinity chromatography. The Western blotting result confirmed that it was IspD (Figure 2D).

### 3.4 M6 is a non-competitive inhibitor for MEP

**Figure 3:**
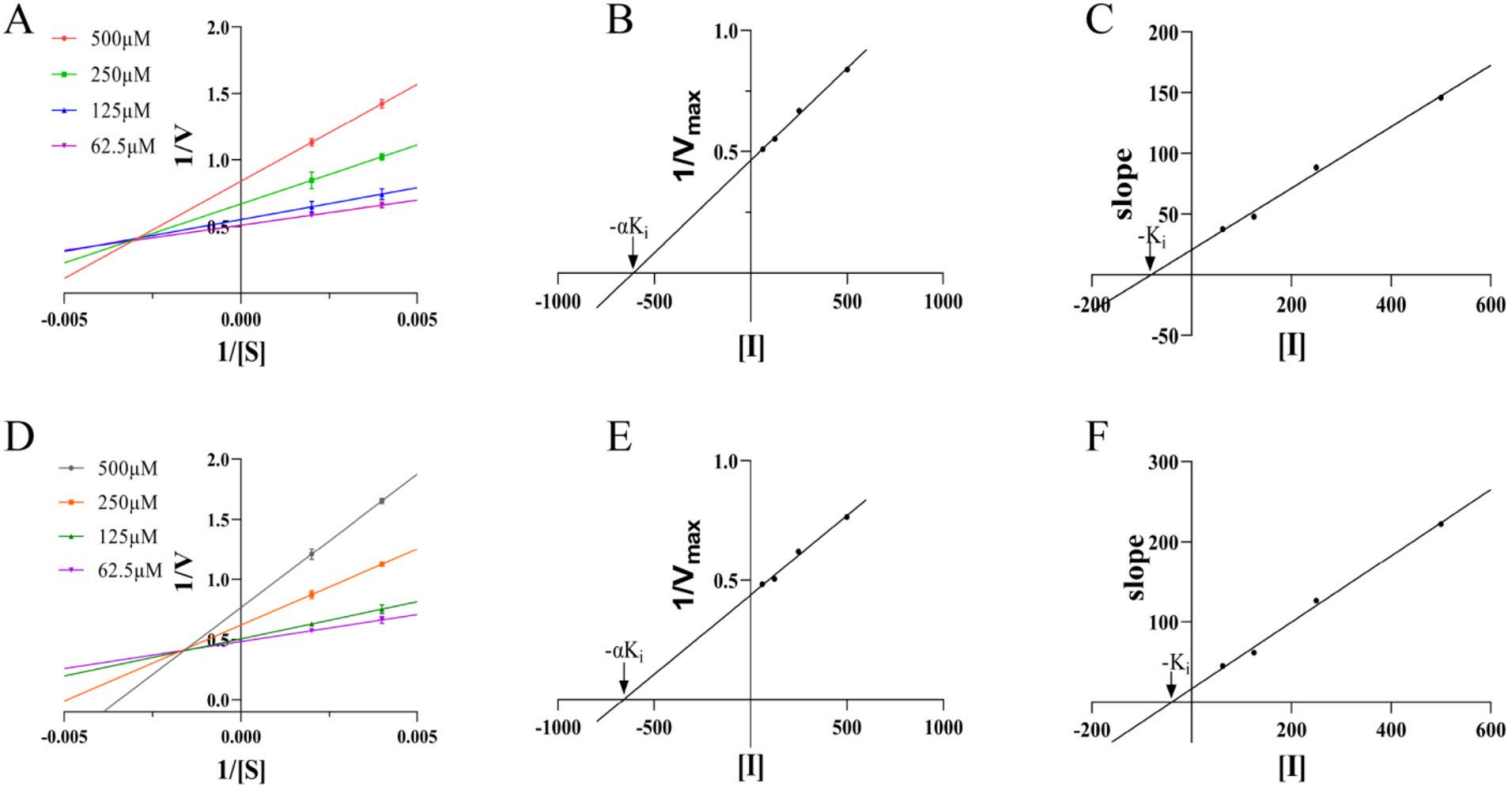
**(A,D)** Double reciprocal method to determine the type of inhibitor. **(B,C,E,F)** Secondary plots for the determination of the inhibitor constants for a noncompetitive inhibitor.

We next attempted to determine the types of M6 inhibitors the double reciprocal method as shown in Figure 3A. The reciprocal of the enzymatic reaction rate (1/v) was plotted against the reciprocal of the substrate concentration (1/S), and multiple inhibitors were used to achieve straight lines with various inhibitor concentrations. The intersection point of the lines was located to the left of the ordinate, indicating that M6 acts as a non-competitive inhibitor of the substrate MEP. By the same method, it is known that M6 acts as a non-competitive inhibitor of the substrate CTP (Figure 3D).When using [I] as the horizontal coordinate and 1/V_max_ as the vertical coordinate, and the value of −αK_i_ is determined from the *x* intercept of the line. The value of −K_i_ is determined from the *x* intercept of a plot of the slope of the lines from the double-reciprocal (Lineweaver-Burk) plot as a function of [I]. The αKi and Ki values of M6 for the substrate MEP are 609.58 μM and 81.33 μM (Figures 3B,C). For CTP, the αKi and Ki values are 657.89 μM and 40.07 μM (Figures 3E,F). This indicates that M6 exhibits stronger inhibition against the substrate CTP compared to the substrate MEP.

### 3.5 Suppression of IspD in *Mycobacterium smegmatis* with CRISPRi inhibits bacterial growth

**Figure 4:**
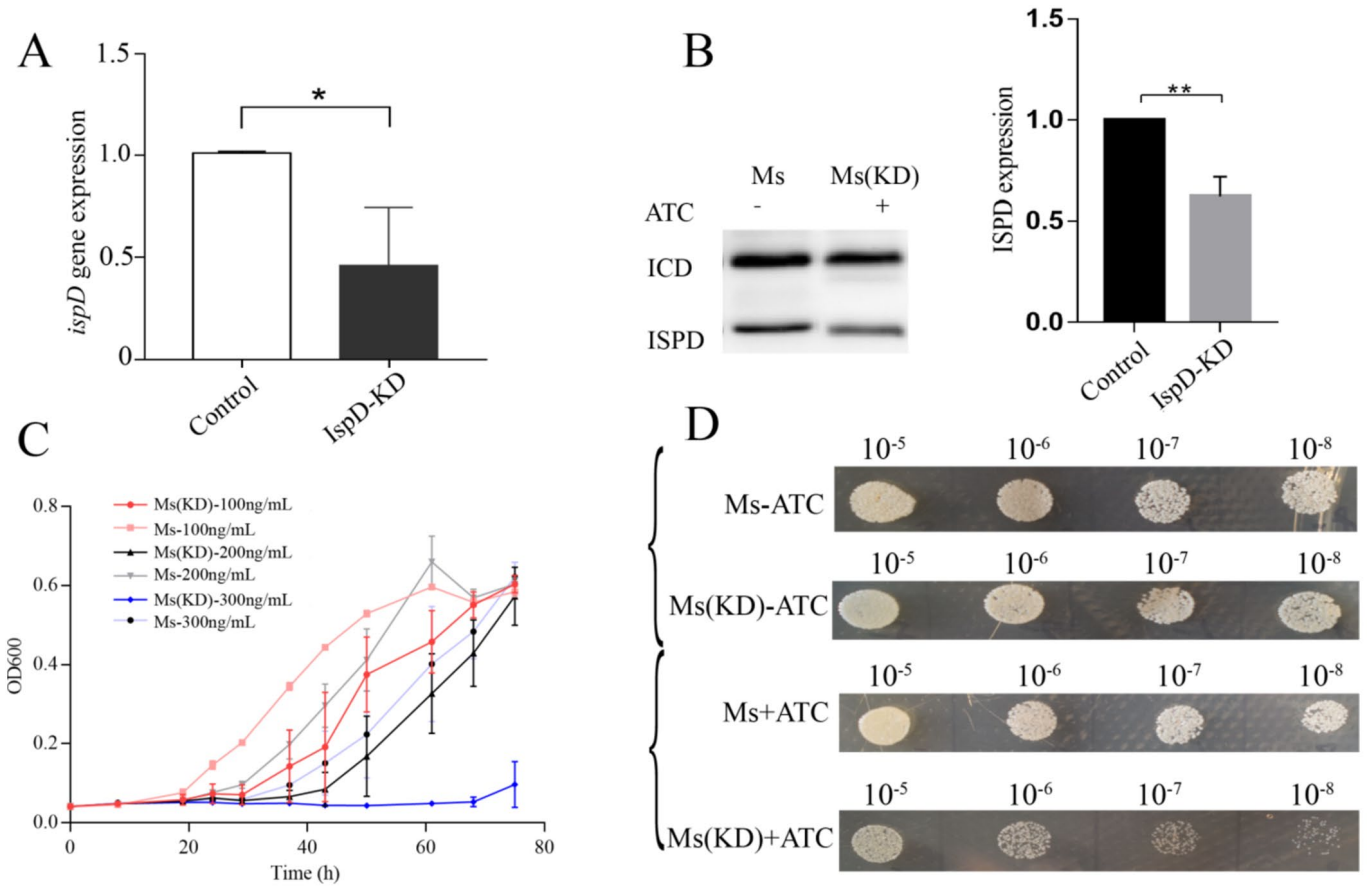
Knockdown of IspD inhibits the growth of *Mycobacterium smegmatis* strain **(A)** Knockdown of ispD gene expression with CRISPRi, *p < 0.05. **(B)** Depletion of IspD protein in IspD bacteria, *p < 0.01. **(C)** Growth curve of the IspD-depleted bacteria induced by tetracycline. **(D)** Slowing growth of stunted plants in solid media.

After electro transformation of *M. smegmatis* receptor cells with plasmid pRH2502 conveying CRISPRi targeting ispD gene, transformed *M. smegmatis* strains were selected by resistance screening. That was, after bacteria were selected in a tetracycline-contained medium, expression of the *ispD* gene from different strains to was first evaluated using qPCR. As shown in Figure 4A, there was a significant decrease in *ispD*, indicating that tetracycline could induce Cas9 expression for suppressing IspD expression in *M. smegmatis*. In addition, Western blot analysis also showed that there was a significant decrease in IspD in the selected *M. smegmatis* IspD (Figure 4B). Importantly, we found that the growth rate of the IspD knockdown strain was significantly inhibited when tetracycline was added into 7H10 agar plates (Figure 4C). The higher the concentration of ATC, the more pronounced the inhibition of the growth rate. At a dilution ratio of 10^–5^, there was an obvious difference. With the dilution ratio increasing, the growth of IspD knockdown strains is significantly suppressed. The growth curve, measured in 7H9 liquid medium, also shows similar results (Figure 4D).

### 3.6 M6 reduces the MIC value of IspD-depleted strains

**Figure 5:**
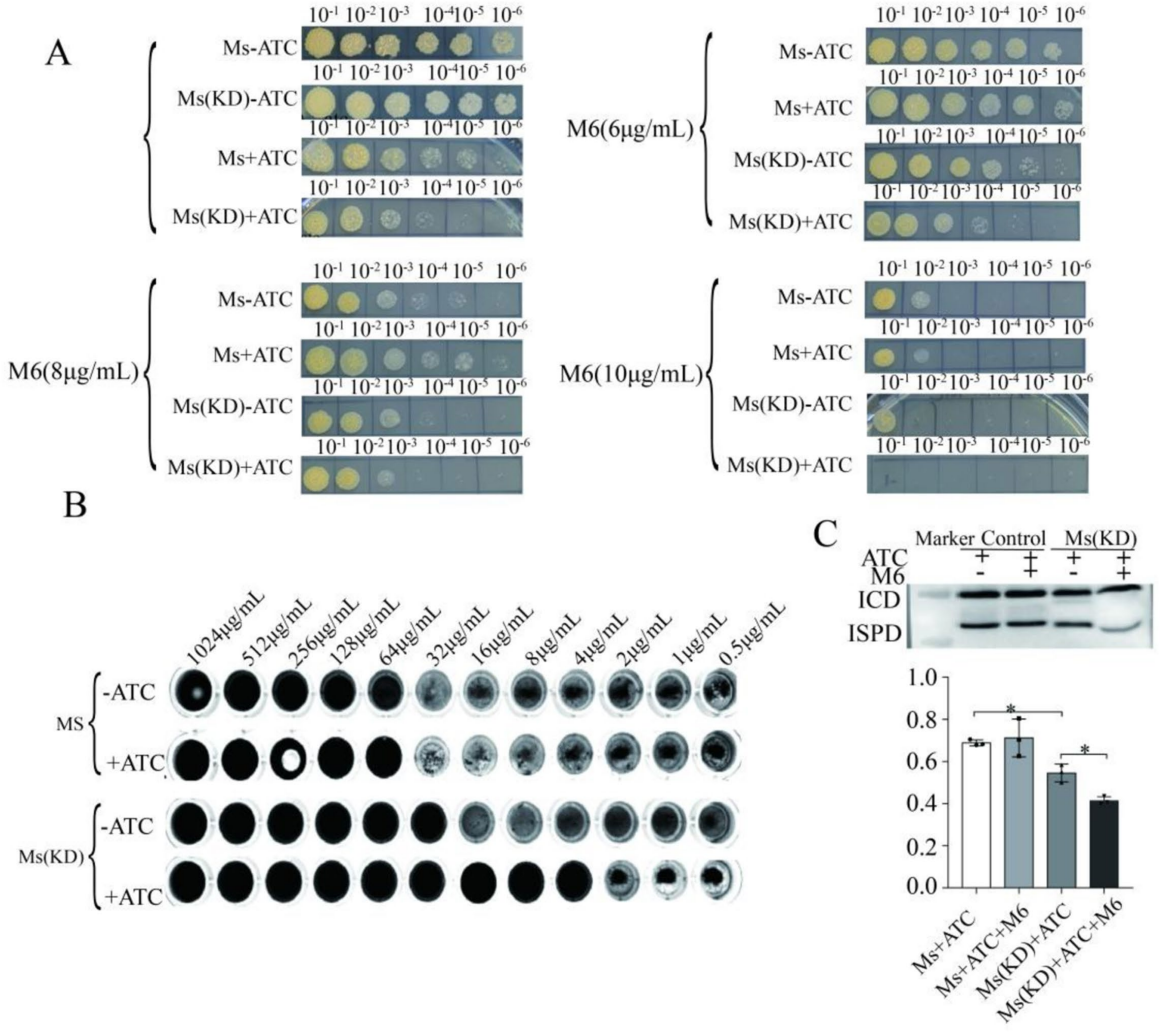
Effect of compound M6 on IspD-knockdown *Mycobacterium smegmatis* model bacteria **(A)** M6 attenuated the growth of *M. smegmatis*_IspD induced by tetracycline. **(B)** MIC of ATC-induced model strains induced by M6. **(C)** M6 further suppressed IspD protein expression in IspD-knockdown *M. smegmatis*. * *p* < 0.05; ATC, Anhydrous tetracycline.

We next evaluated if M6 had any effects on the growth of IspD-knockdown cells. While IspD knockdown inhibited the growth of *M. smegmatis* (Figure 5A), addition of M6 (6, 8, and 10 μg/mL) inhibited the IspD-depleted cells. Notably, with the concentration of M6 increasing, the growth of the IspD knockdown strain was suppressed further.

To identify the target specificity, we measured the MIC values of M6 using 2-fold gradient dilution methods and resazurin stain. The results showed that the MIC value of M6 against *M. smegmatis* with IspD knockdown on the plate decreased from 32 μg/mL to 4 μg/mL under tetracycline induction, indicating an 8-fold decline (Figure 5B).

*Mycobacterium smegmatis* IspD was then cultured until the logarithmic growth phase and then for additional 6 h after diluted in 1:100. The cultures were then subjected to incubation with 100 ng/mL of tetracycline and 10 μg/mL of compound M6 until the logarithmic growth phase of the bacteria was reached. After centrifugation, the bacteria were sonicated to remove the supernatant, and the expression of IspD was verified by Western blot (Figure 5C). The data in Figure 5 also showed that the Tetracycline treatment resulted in a decrease in IspD protein expression, while compound M6 exhibited an even greater ability to lower IspD expression in knockdown strains with suppressed IspD. These results suggest that compound M6 can inhibit bacterial growth by suppressing IspD expression.

## 4 Discussion

Tuberculosis is an infectious disease that poses a serious threat to human health. In recent years, the increasing problem of drug-resistant TB and HIV co-infection has led to the loss of efficacy of conventional anti-TB drugs. In light of the progressively escalating issue of drug-resistant tuberculosis (TB), there is an urgent need to expedite the development of novel anti-TB medications. The structure search of M6 revealed that this compound had no relevant reports on its activity against *MTB* (Palomino, 2013; Deoghare, 2013). However, it was found that para-bromine, which shares a 94% similarity to M6, has biological activity in killing Drosophila eggs. Most of these compounds show strong activity. The preliminary antibacterial activity test revealed that M6 exhibited an 89% inhibitory effect on powdery mildew (live) at a concentration of 10^–3^ g/L. Moreover, it displayed 65, 55, and 50% antibacterial activity against cotton red rot, cotton wilt, and anthracnose respectively, at a concentration of 10^–4^ g/L. These findings strongly suggest that M6 possesses ideal properties as a probe compound for TB research. Based on the drug target research of the anti-tuberculosis drugs bedaquiline (Palomino, 2013; Deoghare, 2013) and delamanid (Deoghare, 2013; Alsayed and Gunosewoyo, 2023), which have received approval for application, it is evident that the discovery of new compounds with anti-tuberculosis activity and the clarification of their mechanism of action are crucial for the development of novel anti-TB drugs and the identification of drug targets.

Thereby in this study, we selected M6 as a probe molecule to identify potential drug targets for anti-TB treatment by molecular docking, and then found that IspD is one of M6 targets in *M. smegmatis* via multiple approaches including enzyme kinetics and CRISPRi.

In the beginning, our objective was to induce mutations and conduct genome sequencing. Nevertheless, after undergoing three rounds of UV mutation screening at a low dosage of M6, strains with a MIC greater than 16-fold were not obtained. This finding indicates that M6 exhibits difficulty in developing resistance in model bacteria. Consequently, it suggests that the mechanism of action of M6 on bacteria is novel and that the target it acts upon is crucial for bacterial functions. We subsequently devised the study protocol using molecular docking techniques. The discovery of the IspD protein through the molecular docking suggests that IspD could potentially be target of M6. IspD is the 2-C-methy1-D-erythriol 4-phosphate cytidylyl-transferase, which is also a key enzyme in the 2-C-methy1-D-erythriol 4-phosphate pathway (MEP) (Putra and Listiani, 2023; Frank and Groll, 2017). In *M. tuberculosis*, isoprene and its derivatives, which are the end products of MEP, play a crucial role as essential precursors for bacterial metabolism and cell wall synthesis. Furthermore, it is worth noting that the MEP pathway is not present in human, animal, or plant cells, which strengthens the likelihood that IspD serves as a highly specific and effective drug target against TB (Banerjee and Sharkey, 2014; Jarchow-Choy et al., 2014). Subsequent studies conducted on enzyme levels *in vitro* and gene inhibition in bacteria have further confirmed that M6 can effectively act on the IspD protein. These findings provide additional evidence that the primary target of M6 is indeed this particular protein.

In the selection aspect of the genetic inhibition research program within bacterial cells, we have opted for the gene interference technology known as Clustered Regularly Interspaced Short Palindromic Repeats (CRISPR) (Hillary and Ceasar, 2023). Among them, CRISPRi (Bendixen and Jensen, 2023) is a gene interference technology comprising a Cas9 protein that retains the binding function while losing the endonuclease cleavage function. In this study, we utilized the dCas9 and sgRNA expression plasmids pRH2502 and pRH2521, which were designed by Singh and Carette (2016). For the purpose of inhibition, the expression of the *M. smegmatis* ispD gene. While pRH2502 and pRH2521, which have tetracycline-induced promoters uv15tetO and Pmyc1tetO, were able to express dCas9 and sgRNA when anhydrous tetracycline was induced, the expression of the IspD protein was reduced. By measuring the tolerance of bacteria to the compound M6, it was determined whether M6 could act on the IspD protein in bacteria and exert antimicrobial activity. Our results confirmed that the minimum inhibitory concentration (MIC) of M6 decreased approximately eightfold, going from 32 μg/mL to 4 μg/mL. This indicates that the bacteria became more sensitive to the drug when there was low expression of the IspD protein. Additionally, the study showed a positive correlation between the concentration of M6 needed to inhibit bacterial growth in *M. smegmatis* and the level of IspD protein expression. In our previous study, it was found that the MIC of M6 for MTB and Corynebacterium glutamate was 0.5 μg/mL. However, the MIC of M6 for *M. smegmatis* and Mycobacterium marine was greater than 20 μg/mL. This difference in MIC may be related to the varying expression levels of the IspD protein in different bacteria. Further studies are required to confirm this.

Taken together, our study has confirmed that the new anti-TB lead compound M6 is the primary target of the IspD enzyme of Mycobacterium *in vitro* and *in vivo*. This finding provides potential candidate lead compound molecules and new drug targets for the further development of new anti-TB drugs.

## Data Availability

The original contributions presented in the study are included in the article/supplementary material, further inquiries can be directed to the corresponding authors.
